# Clinical and Biological Signature of Osteochondritis Dissecans in a Cross-Sectional Study

**DOI:** 10.1155/2018/5458704

**Published:** 2018-05-28

**Authors:** Elena Gabusi, Cristina Manferdini, Francesca Paolella, Laura Gambari, Elizaveta Kon, Giuseppe Filardo, Erminia Mariani, Gina Lisignoli

**Affiliations:** ^1^SC Laboratorio di Immunoreumatologia e Rigenerazione Tissutale, Istituto Ortopedico Rizzoli, Via di Barbiano 1/10, 40136 Bologna, Italy; ^2^Laboratorio RAMSES, Istituto Ortopedico Rizzoli, Via di Barbiano 1/10, 40136 Bologna, Italy; ^3^Department of Biomedical Sciences, Humanitas University, Via Manzoni 113, Rozzano, 20089 Milan, Italy; ^4^Humanitas Clinical and Research Center, Via Manzoni 56, Rozzano, 20089 Milan, Italy; ^5^Clinica Ortopedica e Traumatologica I, Laboratorio NABI, Istituto Ortopedico Rizzoli, Via di Barbiano 1/10, 40136 Bologna, Italy; ^6^DIMEC, Alma Mater Studiorum Università di Bologna, Via Massarenti 9, 40138 Bologna, Italy

## Abstract

The healing potential of knee osteochondritis dissecans (OCD) focal lesions is not well defined. We performed a cross-sectional study correlating local and systemic biological characteristics with the patients' characteristics. We evaluated both local tissue markers (CD34, CD146, CD166, and tartrate-resistant acid phosphatase (TRAP)) and systemic serum biomarkers (fragments or propeptide of type II collagen: C2C, CTX-II, CPII, and TRAP5b) on histologically scored osteochondral fragments or serum from OCD patients. These biological features were associated with the patients' characteristics (IKDC subjective score, age, and body mass index (BMI)). Histological cartilage tissue score correlated with patients' IKDC and C2C and CPII biomarkers. CPII correlated also with histological bone tissue score. The percentage of CD146 positive cells in cartilage and CD34 positive cells in bone highly correlated with the patient's age and BMI, respectively. The percentage of TRAP in bone was directly correlated with both IKDC and age. Multivariate statistical analysis evidenced that only four parameters significantly predicted IKDC. In conclusion, a complete picture of OCD knee characteristics, defined by local and systemic markers of cartilage and bone remodeling, together with the patients' characteristics, might help to better understand the healing potential of each patient and to target and improve current OCD treatments.

## 1. Introduction

Osteochondritis dissecans (OCD) of the joints has been recently defined as a focal idiopathic alteration of subchondral bone which may cause progressive changes in articular cartilage with partial or complete osteochondral detachment [1]. OCD includes two populations of patients: the juvenile form in young adolescent with open physes and the adult form in older adolescent and adults with closed physes [2, 3]. Although its original description dates back to 1887 by Konig, many questions regarding etiology, treatments, and histology remain undefined. Depending on the size of the lesions (<2 cm or >2 cm), OCD is treated using different surgical techniques such as microfractures, osteochondral autografts, and osteochondral allografts or using biomaterials with variable success rate [4, 5].

Different authors have reported that subchondral bone is involved in the etiopathological process of OCD. However, a detailed review on histological and immunohistochemical analysis of OCD fragments [6–9] found that OCD knee histological studies had variable findings and the theory of etiology was based only on a limited and not standardized research in this field. Until now, deep knowledge on OCD etiology has been limited by the considerable variation in the analytic histological techniques used in different studies [9–11]. To shed some light into the healing potential of OCD lesions, an accurate focus on local markers and systemic serum biomarkers of cartilage and bone remodeling, as well as patients' characteristics, is necessary and it could be the basis for better targeting and improving of current OCD therapies [12, 13].

Typical immunohistochemical markers of cartilage and bone remodeling are CD146, CD166, tartrate-resistant acid phosphatase (TRAP), and CD34. CD146 and CD166 are markers used to identify the subpopulation of MSCs progenitor cells located in bone [14] and cartilage [15, 16]. TRAP is a marker used to evaluate how cells participate in the resorption of cartilage matrix or mineralized bone matrix and it is highly expressed in polynucleated osteoclasts and chondroclasts [17], while CD34 is used as marker of endothelial cells for evaluating vascularization: these parameters are directly associated with tissue remodeling.

Among systemic biomarkers of tissue remodeling, C-telopeptide fragments of type II collagen (CTX-II) and collagenase-cleaved fragments of type II collagen (C2C) are widely used as predictive biomarkers of joint degeneration in osteoarthritis (OA). These are often associated with carboxy-terminal propeptide of type II collagen (CPII), a marker of cartilage synthesis, and TRAP5b, a marker of osteoclast activity in bone [18, 19]. Recently, CTX-II has also been investigated [20] in patients with focal cartilage lesion of the knee who showed a higher level compared to healthy subjects.

The aim of this study was to combine, in a cross-sectional study, the evaluation of both local and systemic biomarkers of cartilage and bone remodeling and to associate them with OCD patient characteristics in order to have a more complete picture of this pathology, which could shed some light on the healing potential of OCD lesions.

## 2. Materials and Methods

### 2.1. Patient Characteristics

The OCD patients (*N* = 16) included in the study presented focal lesions (at least 1.5 cm^2^ and less than 4 cm^2^) of the articular surface in otherwise healthy joints (no evidence of other chondral-osteochondral, ligament, meniscus, or synovial lesions), with stable and physiologically aligned knees. X-Ray and MRI surgical indication were confirmed intra-articularly, and patients were staged as grade 3 OCD lesions, according to the ICRS evaluation package [https://www.secot.es/uploads/descargas/formacion/escalas_valoracion/ICRS._TRAUMA_CARTaILAGO.pdf], which includes the International Knee Documentation Committee (IKDC). Knee Examination Form 2000 was administered to assess symptoms and function in daily living activities. This questionnaire looks at 3 categories: symptoms, sports activity, and knee functionality. Scores are obtained by adding up the individual items and then transforming the crude total to a scaled number that ranges from 0 to 100 (representing no symptoms and no limitations with daily activities). The characteristics of each patient included in the study are summarized in Table 1.

OCD lesions were intraoperatively found to be unsuitable for fragment refixation; therefore the injured area was removed and evaluated for this current cross-sectional study, while the defect was reconstructed with the implantation of an osteochondral scaffold. The study was approved by the local ethical committee and, prior to inclusion, all patients signed a written informed consent form.

### 2.2. Histochemical Analysis and Scoring

Osteochondral fragments were fixed in a freshly prepared 9 : 1 mixture of B5 solution (mercuric-chloride containing fixative)/40% formaldehyde and embedded in paraffin and both cartilage and bone tissues were scored as previously described [21]. Briefly, at least 5 serial sections from each osteochondral fragment were stained with Safranin O fast green and both cartilage and bone tissues were histologically scored by two readers (Elena Gabusi and Gina Lisignoli). The maximum cartilage and bone scores were 16 and 10 (highly degenerated tissue), respectively, while normal tissue was scored 0.

### 2.3. Immunohistochemical Analysis

Histological sections were deparaffinized and incubated at room temperature for 1 hour with the following monoclonal mouse anti-human antibodies: CD146 (clone N1238, Novocastra, Leica Biosystems, Newcastle, UK), CD166 (clone MOG/07, Abcam, Cambridge, UK), TRAP (clone 26E5, Novocastra), or CD34 (clone QBEnd, DakoCytomation, Glostrup, Denmark) diluted in TBS containing 0.25% BSA and 0.1% NaN_3_. Negative controls were performed by omitting the primary antibody, and isotype-matched controls were performed by using an isotype-matched primary antibody.

Semiquantitative analysis of immunohistochemistry stained slides was performed on 15 microscopic fields (20x objective lens) for each section. The analysis was performed using Red/Green/Blue (RGB) with Software NIS-Elements and Eclipse 90i microscope (Nikon Instruments Europe BV) equipped with a CCD camera (dimension of the sensor: 2/3 inches) mounted on 0.7x C-mount. Briefly, we acquired the total number of blue-stained nuclei and the total number of positive-stained red cells in each field (358 × 269.15 *μ*m). Data were expressed as a mean percentage of positive cells for CD146, CD166, and TRAP, respectively. The number of positive CD34 vessels was counted manually and expressed as the mean number of vessels/area. All data obtained from each fragment/section were then expressed as median and 10–90 percentiles.

### 2.4. Biomarkers of Cartilage and Bone Remodeling

On the same day of surgery, a serum sample was collected from each patient. The following serum biomarkers of cartilage remodeling were analyzed following the company instructions: CTX-II (Elabscience Biotechnology Co., Ltd., Wuhan, Hubei Province, China), C2C (IBEX Pharmaceutical Inc., Montréal, Québec, Canada), CPII (IBEX Pharmaceutical Inc., Montréal, Québec, Canada), and bone remodeling TRAP5b (Quidel Corporation, Athens, OH, USA). Control healthy subjects (*N* = 8) were also evaluated. Data were expressed for all assays as ng/ml, except TRAP5b that was expressed in U/ml.

### 2.5. Statistical Analysis

The normal distribution of continuous data was analyzed with Kolmogorov-Smirnov test and since data were not normal, we used nonparametric tests. Statistical analysis for comparing cartilage and bone was performed with Mann–Whitney *U* test for unpaired two-group data. Spearman correlations were evaluated between clinical (IKDC subjective score) and biological (cartilage score or bone score or immunohistochemical markers or serum biomarkers) markers. We performed multivariate analysis using the Generalized Linear Model (GLM) based on gamma distribution to assess the variables that significantly and independently predicted the IKDC subjective score, and the Wald method was used for the selection of the variables.

CSS Statistical Software (StatSoft Inc., Tulsa, OK, USA) was used for analysis. All results were considered significant for *p* < 0.05.

## 3. Results

### 3.1. Histological Characterization of OCD Fragments

**Figure 1:**
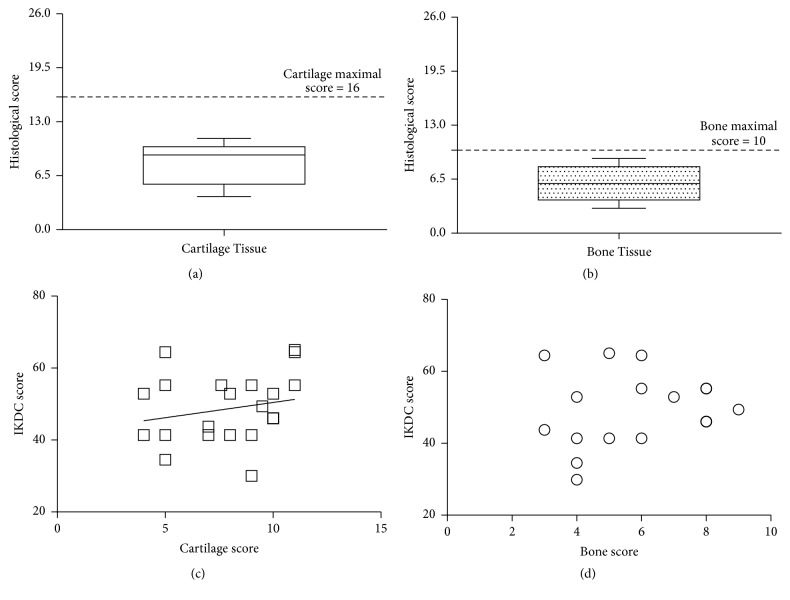
*Cartilage and bone histological scores correlations with patient characteristics*. (a)-(b) Cartilage and bone scores are represented as box plot with median and 25th and 75th interquartile ranges. Cartilage maximal score: 16; bone maximal score: 10. Normal tissue = 0. (c)-(d) Correlation between cartilage and bone scores and IKDC subjective score of each patient. Each point represents the histological cartilage or bone score performed on the different fragments (*N* = 23).

Osteochondral fragments were obtained from 16 patients (4 females and 12 males, Table 1) with lesions mainly in the medial femoral condyle (81.3%). Safranin O-stained sections evidenced that all patients showed a median cartilage score of 9 (25th percentile = 5.5 and 75th percentile = 10) with a maximal score equal to 16 (Figure 1(a)), while bone score showed a median of 6 (25th percentile = 4 and 75th percentile = 8) with a maximal score equal to 10 (Figure 1(b)).

### 3.2. Cartilage Tissue Score Correlated with Patient IKDC Subjective Score

Further analysis was performed to determine if cartilage and bone tissue scores were correlated with the patient's characteristics (IKDC subjective score, age, and BMI). We found that only cartilage tissue score was significantly correlated (Rho = 0.412; *p* = 0.05) with the IKDC subjective score (Figure 1(c)), while there was no correlation with bone score (Figure 1(d)). No correlation was also found with age or BMI (data not shown).

### 3.3. Different Levels of Systemic Biomarkers in OCD Compared to Healthy Subjects

**Figure 2:**
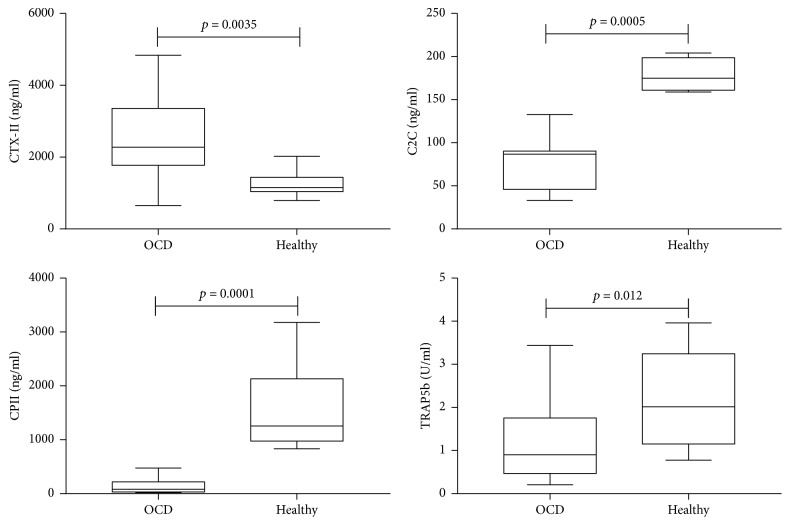
*Serum biomarkers evaluation in OCD and healthy subjects*. Biomarkers of cartilage degradation (CTX-II and C2C), synthesis (CPII), and bone remodeling (TRAP5b) are represented as box plot with median and 25th and 75th interquartile ranges. CTX-II, C2C, and CPII were expressed as ng/ml, while TRAP5b is expressed as U/ml.

Systemic biomarkers of cartilage degradation (CTX-II and C2C) or synthesis (CPII) and bone remodeling (TRAP5b) were measured in OCD patients and compared to healthy subjects. We found that CTX-II was significantly higher in OCD (*p* = 0.0035) compared to healthy subjects, while C2C, CPII, and TRAP5b were significantly decreased in OCD (*p* = 0.0005, *p* = 0.0001, and *p* = 0.012, resp.) compared to healthy subjects (Figure 2). We therefore investigated whether these biomarkers of cartilage and bone remodeling were associated with the patients characteristics (IKDC subjective score, age, and BMI).

### 3.4. Cartilage and Bone Tissue Scores Differently Correlated with Serum Biomarkers

**Figure 3:**
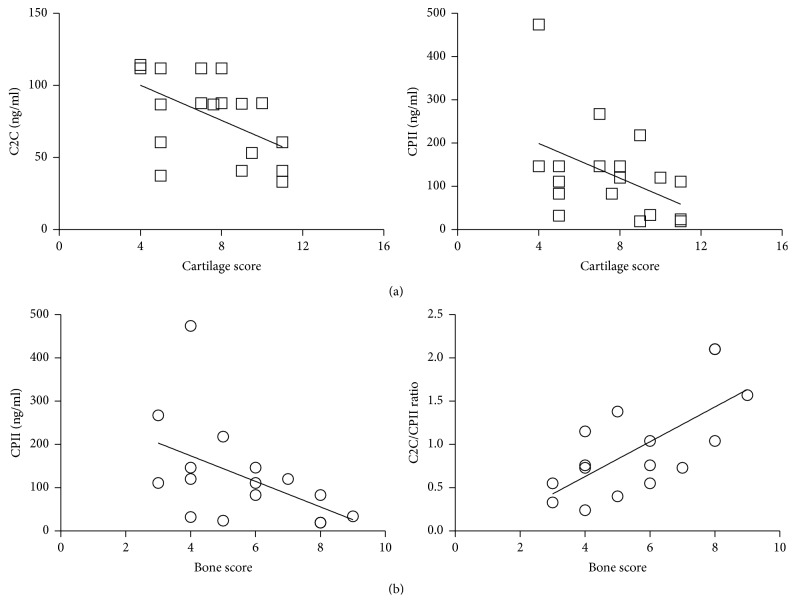
*Correlation between biomarkers and cartilage or bone histological score*. (a) Correlation between C2C and CPII and cartilage histological score. Each point represents the value of the biomarker referred to the cartilage score performed on the different fragments. (b) Correlation between CPII and C2C/CPII ratio and bone histological score. Each point represents the value of the biomarker referred to bone score performed on the different fragments.

The correlation of both cartilage and bone tissue scores with these biomarkers evidenced a significant inverse correlation only for C2C (Rho = −0.582; *p* = 0.009) and CPII (Rho = −0.528; *p* = 0.02) with cartilage tissue score (Figure 3(a)), showing lower levels of these biomarkers in patients with higher degraded cartilage. Interestingly, no correlation was found with CTX-II or cartilage biomarkers ratios (C2C/CPII and CTX-II/CPII) or TRAP5b biomarkers (data not shown). By contrast, bone tissue score showed a significant inverse correlation with CPII (Rho = −0.491; *p* = 0.033) and a direct correlation with C2C/CPII ratio (Rho = 0.581; *p* = 0.009) (Figure 3(b)) but it was not correlated with bone degradation biomarker TRAP5b or with CTX-II and C2C.

### 3.5. Immunohistochemical Markers of Tissue Remodeling Were Highly Expressed in Bone

**Figure 4:**
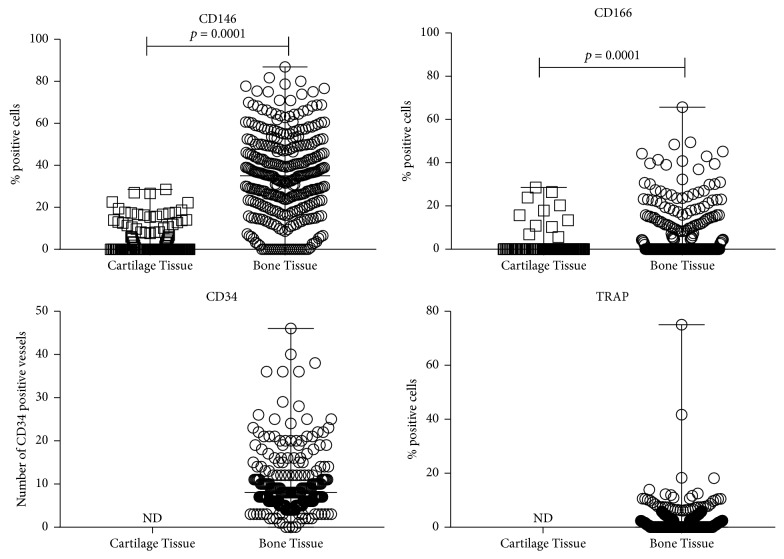
*Immunohistochemical analysis of CD146, CD166, CD34, and TRAP on cartilage and bone tissues*. Each point represents the analysis performed in each field of the analyzed section. CD146, CD166, and TRAP are expressed as percentage of positive cells; CD34 is expressed as number of positive vessels.

To define osteochondral tissue remodeling in OCD fragments, we evaluated the following markers with immunohistochemistry in both cartilage and bone tissues: CD146, CD166, CD34, and TRAP. As shown in Figure 4, all tested markers showed a significantly (CD146 and CD166, *p* = 0.0001) higher percentage of positive cells located mainly in bone rather than in cartilage.

### 3.6. CD146, CD166, CD34, and TRAP Tissue Markers Were Differently Correlated with Histological Scores and Serum Biomarkers

We also investigated whether CD146, CD166, CD34, and TRAP were linked to cartilage and bone scores or to cartilage and bone remodeling biomarkers. As shown in Table 2, an inverse correlation between percentage of cartilage cells positive to CD146 and CD166 and cartilage score was observed. Interestingly, a direct correlation with both degradation (CTX-II and C2C) and synthetic (CPII) cartilage biomarkers was found with the percentage of CD146 cells. By contrast, the percentage of CD166 positive cells in cartilage was inversely correlated with both CTX-II and C2C.

The percentage of TRAP positive cells in bone was directly correlated with the bone score (Rho = 0.125; *p* = 0.039).

### 3.7. Immunohistochemical Markers of Cartilage and Bone Remodeling Were Differently Correlated with the Patients' Characteristics

We correlated all cartilage and bone remodeling immunohistochemical markers with IKDC subjective score, age, and BMI. As shown in Table 3, the percentage of CD146 in cartilage and the percentage of CD34 in bone were indirectly correlated with age and BMI, respectively. Interestingly, the percentage of TRAP in bone was directly correlated with both IKDC subjective score and age.

### 3.8. Specific Systemic and Local Markers as a Model to Predict IKDC Subjective Score

**Figure 5:**
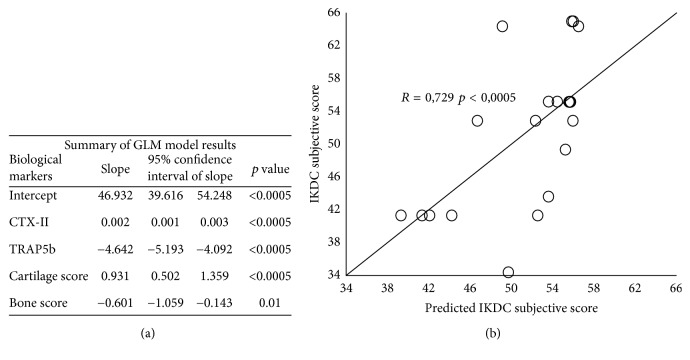
*IKDC subjective score prediction*. (a) GLM model results evidence that four of the analyzed parameters (CTX-II, TRAP5b, cartilage score, and bone score) are good predictors of IKDC subjective score. (b) Correlation between IKDC subjective score and predicted IKDC subjective score (*R* = 0,729; *p* < 0.0005). Each point represents the value of IKDC subjective score and its predicted value calculated on GLM model results.

To define which of the significant biological markers (cartilage score, bone score, percentage of CD146 and CD166 in cartilage, percentage of CD34 in cartilage or bone, percentage of TRAP in bone, CTX-II, C2C, CPII, TRAP5b, C2C/CPII, and CTX-II/CPII), analyzed by Spearman correlation, was the best predictor of IKDC subjective score, the multivariate GLM test was performed. As shown in Figure 5(a), we found that only cartilage and bone score together with CTX-II and TRAP5b were biological markers that significantly and independently predicted IKDC subjective score. In particular, the predicted values, calculated using the GLM model results (Figure 5(a)), had a high correlation with the IKDC subjective score (*r* = 0.729; *p* < 0.0005) (Figure 5(b)).

## 4. Discussion

OCD of the knee is a common and poorly understood pediatric and adult condition. Decision regarding different surgical treatments remains unsupported by current literature [1, 12, 13]. We concentrated on focal OCD of the knee by performing a cross-sectional study that took into consideration local markers as well as systemic cartilage and bone remodeling biomarkers to assess whether the healing potential was correlated with the patient characteristics (IKDC subjective score, age, and BMI).

Firstly, we confirmed that OCD bone tissue, as recently published [21], showed a higher median histological score compared to cartilage, indicative of active tissue remodeling. The OCD cartilage median score also well reflected the modifications of this tissue and suggested that the focal degeneration of the cartilage matrix could originate from subchondral bone. This observation, based on the use of specific scores for cartilage and bone tissue, has also been confirmed in other previous studies [9, 11].

Moreover, a higher number of cells positive for remodeling markers (CD146, CD166, TRAP, and CD34) have been found in bone than in cartilage, confirming active bone remodeling as previously reported by some histological studies [10] and our recent paper [21]. Histological and immunohistochemical results clearly support the recent definition of OCD as “focal, idiopathic alteration of subchondral bone with risk for instability and disruption of adjacent articular cartilage” [22].

We found that bone histological score is directly correlated only with the percentage of TRAP, a specific enzyme produced by osteoclasts [23]. TRAP was also directly correlated with both IKDC subjective score and age (Table 3), confirming high bone remodeling activity in OCD patients. In fact, it is well known that age plays a decisive role in OCD development and it is important for its prognosis [24]. Moreover, we have also demonstrated that the percentage of CD34 (marker of vascularization) positive cells in bone was significantly negatively correlated with BMI, indicating a lower number of vessels in obese patients, which might suggest a different pathway of bone tissue remodeling.

It is well known that the percentage of markers of cartilage derived mesenchymal stem cells (CD146 and CD166) significantly decreases in worst histological cartilage score and our data also confirmed lower remodeling of cartilage tissue [15, 16]. In particular, although the decrease of CD146 percentage did not reach a significant correlation with cartilage score (*p* = 0.59), data showed a correlation with aging, confirming that cartilage remodeling is also strictly dependent on the age of the patients. Interestingly, the percentage of CD146 positive cells increased with both biomarkers of cartilage degradation (CTX-II and C2C) and synthesis (CPII), while CD166 was inversely correlated, indicating that the active process of cartilage remodeling was also influenced by systemic factors.

It has been shown that these serum systemic biomarkers are good surrogates because they reflect the concentrations observed in the synovial fluid from the knee [25]. We have found that systemic biomarkers C2C and CPII are both significantly and inversely correlated with cartilage score, indicating that worst histological characteristics of this tissue corresponded to lower levels of both biomarkers of cartilage degradation and synthesis. It has been shown that CTX-II is correlated with histological Mankin score in osteoarthritis animal model [19] and with severity of knee OA [26]. However, we could not find any correlation between CTX-II biomarkers and cartilage score, although this biomarker could be considered a good indicator of cartilage degradation [25]. Interestingly, Røtterud et al. [20, 23] included in their study both traumatic and OCD patients and correlated CTX-II biomarker with the patients' characteristics. By contrast, our study has focused only on OCD patients and has considered at the same time both local (histological score and immunohistochemical markers) and systemic serum biomarkers to have a complete picture of this disease. Moreover, in line with previously published data [20], we detected CTX-II as the only biomarker in higher amount in OCD patients compared to healthy subjects.

We have found that CPII and C2C/CPII ratio, good indicators of collagen type II synthesis and degradation, are also, respectively, negatively and positively correlated to bone histological score. These data indicate that their levels are associated with worst bone histology with a clear decrease of the synthetic CPII biomarker and an increase of C2C/CPII ratio, confirming the presence of concomitant bone and cartilage remodeling in OCD patients.

The IKDC subjective score is used by clinicians to assess symptoms and functionality in daily living activities: we have found that it is significantly correlated only with cartilage histological score, indicating that in OCD patients (with focal lesions) a better clinical score does not directly correspond to a good histological characteristic of cartilage. This result might suggest that histological signs of cartilage damage occur before the onset of clinical symptoms.

Finally, we have used a GLM model to define which of the analyzed biological markers should be good parameters to estimate the IKDC subjective score and the model has evidenced that only four (histological cartilage and bone scores, CTX-II, and TRAP5b) were good independent predictors. Interestingly, two of them are local (histological cartilage and bone scores) and two are systemic (CTX-II and TRAP5b) biological markers, specific of cartilage or bone, respectively, confirming again that this disease is dependent on balanced remodeling of both bone and cartilage tissues. This GLM model has strongly established a direct link between the four predictors and the IKDC subjective score, suggesting that this could also be a useful predictive tool during the clinical follow-up. However, this hypothesis must be confirmed in a future longitudinal study.

## 5. Conclusions

In conclusion, this study gives an overall picture of knee OCD focal lesions, since we have considered both local and systemic markers of bone and cartilage remodeling and combined them with patients' characteristics to assess their healing potential. These data have allowed us to develop a good GLM predictor model, which might help to better focus on and improve currently used focal knee OCD therapies.

## Figures and Tables

**Table 1:** OCD patients' characteristics.

Patients (*n*.)	16

Lesion location	1 Trochlea
	2 LFC
	13 MFC

Sex	4 Females
	12 Males

IKDC clinical score	55.5 ± 13.4^*∗*^

Age	23 ± 8.3^*∗*^

BMI	24 ± 3.9^*∗*^

Physes	1 open
	15 closed

LFC: lateral femoral condyle, MFC: medial femoral condyle, IKDC: international knee documentation committee, BMI: body mass index. ^*∗*^Mean  ±  SD.

**Table 2:** Correlations between cartilage markers CD146 and CD166 and cartilage score or biomarkers.

	% CD146		% CD166	
	Rho	*p*	Rho	*p*
Cartilage score	−0.449	<0.0005	−0.244	0.009
CTX-II	0.286	<0.0005	−0.441	<0.0005
C2C	0.277	<0.0005	−0.206	0.033
CPII	0.232	0.004	−0.136	0.161

**Table 3:** Correlations between percentage of CD146, CD166, and CD34 in bone and cartilage and patients characteristics.

	% CD146 in cartilage		% CD34 in bone		% TRAP in bone	
	Rho	*p*	Rho	*p*	Rho	*p*
IKDC score	−0.054	0.470	−0.002	0.976	0.191	<0.0005
Age	−0.420	<0.0005	0.063	0.317	0.191	<0.0005
BMI	−0.157	0.082	−0.270	<0.0005	0.095	0.082
